# Specific Frontostriatal Circuits for Impaired Cognitive Flexibility and Goal-Directed Planning in Obsessive-Compulsive Disorder: Evidence From Resting-State Functional Connectivity

**DOI:** 10.1016/j.biopsych.2016.08.009

**Published:** 2017-04-15

**Authors:** Matilde M. Vaghi, Petra E. Vértes, Manfred G. Kitzbichler, Annemieke M. Apergis-Schoute, Febe E. van der Flier, Naomi A. Fineberg, Akeem Sule, Rashid Zaman, Valerie Voon, Prantik Kundu, Edward T. Bullmore, Trevor W. Robbins

**Affiliations:** a Departments of Psychology, University of Cambridge, Cambridge; b Psychiatry, University of Cambridge, Cambridge; c Behavioural and Clinical Neurosciences Institute, University of Cambridge, Cambridge; d Brain Mapping Unit, University of Cambridge, Cambridge; e Hertfordshire Partnership University National Health Service Foundation Trust, Hertfordshir; f University of Hertfordshire, Hertfordshire; g Cumbria Partnership National Health Service Foundation Trust, National Health Service, Penrith, Cumbria, United Kingdom; h Brain Imaging Center and Translational, Icahn Institute of Medicine at Mt. Sinai, New York, New York; i Molecular Imaging Institute, Icahn Institute of Medicine at Mt. Sinai, New York, New York

**Keywords:** Cognitive flexibility, Frontostriatal circuits, Functional connectivity, Goal-directed planning, Obsessive-compulsive disorder, Resting state

## Abstract

**Background:**

A recent hypothesis has suggested that core deficits in goal-directed behavior in obsessive-compulsive disorder (OCD) are caused by impaired frontostriatal function. We tested this hypothesis in OCD patients and control subjects by relating measures of goal-directed planning and cognitive flexibility to underlying resting-state functional connectivity.

**Methods:**

Multiecho resting-state acquisition, combined with micromovement correction by blood oxygen level–dependent sensitive independent component analysis, was used to obtain in vivo measures of functional connectivity in 44 OCD patients and 43 healthy comparison subjects. We measured cognitive flexibility (attentional set-shifting) and goal-directed performance (planning of sequential response sequences) by means of well-validated, standardized behavioral cognitive paradigms. Functional connectivity strength of striatal seed regions was related to cognitive flexibility and goal-directed performance. To gain insights into fundamental network alterations, graph theoretical models of brain networks were derived.

**Results:**

Reduced functional connectivity between the caudate and the ventrolateral prefrontal cortex was selectively associated with reduced cognitive flexibility. In contrast, goal-directed performance was selectively related to reduced functional connectivity between the putamen and the dorsolateral prefrontal cortex in OCD patients, as well as to symptom severity. Whole-brain data-driven graph theoretical analysis disclosed that striatal regions constitute a cohesive module of the community structure of the functional connectome in OCD patients as nodes within the basal ganglia and cerebellum were more strongly connected to one another than in healthy control subjects.

**Conclusions:**

These data extend major neuropsychological models of OCD by providing a direct link between intrinsically abnormal functional connectivity within dissociable frontostriatal circuits and those cognitive processes underlying OCD symptoms.

Persistent intrusive thoughts and repetitive actions characterize the symptoms of obsessive-compulsive disorder (OCD). Over and above these often highly specific clinical symptoms is a general tendency toward poor performance on tasks requiring flexible behavior (1, 2, 3). Preclinical and clinical evidence indicates the integrity of the basal ganglia and their connections with the frontal cortex to be crucial in enabling the affective, cognitive, and motor flexibility necessary for goal-directed behavior (4, 5). These data are thus broadly consistent with a leading neural model implicating frontostriatal circuits in the pathogenesis of OCD (6, 7, 8). However, this model has not been clearly linked to underlying cognitive or psychological processes mediated by this circuitry. This link has recently been provided by a major hypothesis concerning OCD (9, 10), which postulates that OCD symptoms arise from impairments in goal-directed behavior, leading to autonomous habitual behavior as a consequence of functional imbalances in frontostriatal circuitry (11, 12).

Resting-state activity derived from functional magnetic resonance imaging (fMRI) enables inference of the strength of functional connectivity between different brain regions. OCD patients exhibit hyperconnectivity of ventrolimbic corticostriatal regions that correlates with symptom severity as well as hypoconnectivity of the caudate and putamen with cortical areas (13). However, the behavioral significance of this pattern of functional connectivity is yet to be established.

Here, we focused on attentional set-shifting (cognitive flexibility) as well as goal-directed planning. Deficits in these executive functions represent candidate endophenotypes for the disorder (14, 15) and have been related to OCD symptoms (16). Planning is measured as the ability to attain a goal via a prepared series of actions controlled by a single instrumental contingency (17). OCD patients are impaired in this goal-directed capacity, especially at more demanding levels (18). Compared with control subjects, OCD patients exhibit decreased functional activation of the dorsolateral prefrontal cortex (PFC), caudate, and putamen during planning (19), consistent with other behavioral data suggesting an imbalance between the control exerted by goal-directed and habit systems over behavioral output in OCD (12, 20).

We hypothesized that impairment of frontostriatal circuitry entailing caudate and putamen and separate regions of PFC likely disrupts implementation of flexible goal-directed behavior in OCD patients. Notwithstanding overlap (21), the existence of topographic projections of different PFC regions to striatal regions in monkeys (4, 21) and humans (22) suggests that separate frontostriatal circuits are implicated in regulating cognitive control and aspects of executive functioning (4). Thus, a lateral circuit including the ventrolateral PFC has been suggested to mediate switches in behavioral set (23, 24, 25) with a dorsolateral PFC circuit being related to goal-directed planning (26). On the basis of its PFC inputs, the ventral striatum has alternatively been implicated in affective control and reward processing (27).

We used resting state to investigate functional connectivity in OCD patients and to overcome limitations of task-related studies in which case-control differences in activation might be the result of task performance, effort, or strategy rather than the reflection of underlying core abnormalities. Moreover, the topography of networks arising from synchronous spontaneous functional activity is compatible with the underlying structural connectivity and consists of regions known to share common functions (28). We leveraged on a novel multiecho acquisition method with significantly increased signal-to-noise ratio and two complementary analytical approaches. First, we formulated specific a priori hypotheses and used seed-based analysis to test whether dysregulated resting-state connectivity arising from caudate and putamen accounted for impairments in cognitive flexibility and executive planning. We hypothesized that if functional abnormalities in these circuits were relevant to OCD, then we should find specific patterns of altered connectivity being associated with cognitive flexibility and goal-directed planning in patients. Consistent with previous evidence (23, 24, 25, 26), we predicted that ventrolateral and dorsolateral PFC circuits would mediate cognitive flexibility and goal-directed planning, respectively. To overcome the limitation of exclusively focusing on a priori regions, the second approach applied a whole-brain data-driven graph theoretical analysis to identify novel features of abnormal brain network organization in OCD patients (29).

## Methods and Materials

### Participants

The study included 87 participants, consisting of 44 OCD patients and 43 healthy control subjects matched for relevant demographic variables (Table 1, Supplement). The OCD sample included 27 medicated and 17 unmedicated patients; all but one of the medicated patients were taking selective serotonin reuptake inhibitors (Supplement). OCD patients reported higher levels of depressive symptoms and anxiety, although well below clinical threshold (Table 1). Unless otherwise reported, there were no differences between medicated and unmedicated OCD patients in the results presented.

**Table 1:** Demographic and Clinical Characteristics and Imaging Motion Assessment of the Studied Sample

Variable	CTL (*n* = 43)	OCD (*n* = 44)	Statistic			
			*χ*^2^	*t*	*df*	*p* Value
Demographic and Clinical Characteristics						
Sex, male/female	22/21	21/23	0.103		1	.749
Hand, right/left	38/5	38/6	0.079		1	.778
Age, years	37.51 ± 12.05	36.14 ± 10.71		0.563	85	.575
Education, years	16.49 ± 3.81	15.77 ± 3.21		0.948	85	.346
Estimated verbal IQ^a^	115.18 ± 6.14	112.73 ± 7.20		1.704	85	.092
OCI-R	4.56 ± 4.34	33.64 ± 11.79		−15.198	85	.000
MADRS	0.77 ± 1.32	8.41 ± 5.29		−9.189	85	.000
STAI-state	26.95 ± 7.83	42.16 ± 10.31		−7.734	85	.000
STAI-trait	33.23 ± 7.74	54.98 ± 8.96		−12.103	85	.000
Y-BOCS total	—	22.00 ± 5.31				
Y-BOCS obsessions	—	10.95 ± 3.22				
Y-BOCS compulsions	—	11.02 ± 2.66				
Onset, years	—	13.39 ± 7.63				
Age at diagnosis, years	—	24.02 ± 7.09				
Duration of disease, years	—	12.11 ± 9.44				
Imaging Motion Assessment						
FD	0.11 ± .05	0.13 ± 0.09		−1.873	85	.065
Motion, high/low^b^	19/24	24/20	0.934		1	.333
BOLD components	23.05 ± 6.42	24.68 ± 8.01		−1.050	85	.297

Values are mean ± SD or *n/n*.

BOLD, blood oxygen level–dependent; CTL, control subjects; FD, framewise displacement; MADRS, Montgomery–Åsberg Depression Rating Scale (59); OCD, obsessive-compulsive disorder patients; OCI-R, Obsessive-Compulsive Inventory-Revised (60); STAI, State-Trait Anxiety Inventory (61); Y-BOCS, Yale-Brown Obsessive Compulsive Scale (62).

a Estimated verbal IQ was measured with the National Adult Reading Test.

b Median split of the main cohort of 87 subjects according to a measure of total motion computed as the sum of FD (32).

### Procedure

#### Imaging Procedure

For resting-state data acquisition, we used multiecho planar sequence with improved signal-to-noise ratio (see Supplement for imaging variables). Participants were instructed to lie quietly with their eyes open and attend to a centrally presented white fixation cross on a black projection screen for 10 minutes; we monitored their degree of alertness by asking to complete the Stanford Sleepiness Scale (30) ruling out differences in levels of arousal across participants (Supplement).

#### Behavioral Testing Procedure

To elucidate the behavioral significance of the functional abnormalities within frontostriatal circuits in OCD patients, in a separate session outside the scanner, the same participants were tested with objective and well-validated CANTAB paradigms. We used the intra-/extradimensional set shift (IED) and the One Touch Stockings of Cambridge (OTS) to measure cognitive flexibility and goal-directed planning, respectively. The IED is a nine-stage task, and the rule for correct responding is modified at the start of each stage. For the IED, crucial stages are the intradimensional shift (IDs) testing for the ability to generalize a rule to new stimuli and the extradimensional shift (EDs) testing cognitive flexibility as the ability to shift attention to a previously irrelevant dimension. On the OTS, planning abilities are tested at different difficulty levels with problem difficulty varying from 1 to 6 moves (see Supplement for description of the paradigms).

### Image Preprocessing

Imaging data were preprocessed and analyzed using Analysis of Functional NeuroImages (AFNI) software (31). To denoise the data, we used a novel integrated procedure taking advantage of multiecho acquisition in combination with Multi-Echo Independent Component Analysis (ME-ICA) (AFNI tool meica.py, version 2.5 beta10) (32). The rationale behind ME-ICA is to classify sources of variance in the fMRI time-series scaling linearly with echo-time and thus confidently regarded as indicative of blood oxygen level–dependent (BOLD) contrast (Supplement). The retained independent components, representing BOLD contrast, were optimally recomposed and visually inspected (see Supplemental Figure S1 for a representative subject). According to multiple, complementary indices, patients and control subjects did not differ for movement in the scanner; there were no significant groups differences in the number of high- versus low-motion subjects in each group or in the number of BOLD components retained or motion as measured by framewise displacement (Table 1).

### Data Analysis

#### Behavioral Analysis

On the IED task, dependent measures at each stage were the number of subjects passing and the number of errors. Data were square root-transformed to stabilize variance and to reduce skewness in the distribution. On the OTS task, we measured the mean number of attempts made before obtaining the correct solution for easy (1–3 moves) and hard (4–6 moves) levels of difficulty (significance threshold, *p* < .025) (18). Data were statistically analyzed using χ^2^, analysis of variance, Student’s *t* test, and the Mann-Whitney *U* test to detect group differences between control subjects and OCD patients. Only the 44 OCD patients were included for Pearson’s correlations between clinical scales and measures of tasks yielding significant group differences.

#### Image Analysis

We tested differences in functional connectivity strength between OCD patients and control subjects from a priori anatomical regions of interest based on the known neurobiological profile of OCD and previous findings in the literature (11, 13). We examined connectivity from the dorsal caudate (DCd), putamen (PUT), and nucleus accumbens (NAc), defined in both hemispheres as 3-mm radial spheres located at Montreal Neurological Institute coordinates automatically provided by the AFNI-supplied atlas, namely, DCd: ± 12 6 14 [labeled as the body of the caudate and analogous to the dorsal striatal seed previously described in the literature (13)]; PUT: ± 24 0 3; NAc: ± 12 8 −8.

Based on evidence of caudate and putamen involvement in executive functions, we tested the a priori hypothesis that dysregulated functional connectivity from the DCd and PUT selectively accounted for impairment on relevant cognitive domains in OCD patients. For the OCD patients only, we used number of errors on the EDs stage and mean number of problems attempted at the hardest level of difficulty (6 moves) of the OTS task as a covariate of interest, to identify brain regions for which significant connectivity with DCd and PUT was significantly related to cognitive flexibility and goal-directed planning. Post hoc analyses investigated NAc connectivity relation with cognitive performance and clinical scores.

ME-ICA denoised data were entered in 3dGroupInCorr to estimate functional connectivity: time-series were extracted from each dataset averaging locally per the seed’s radius and connectivity maps computed with Pearson’s correlation; Fisher’s *r*-to-*z* transform for the appropriate degrees of freedom (i.e., number of BOLD components identified for each subject) was used to derive standard scores. Whole-brain analyses were conducted in combination with cluster-based correction. We applied voxel-level height threshold of *p* < .01 and used 3dClustSim to determine the corrected *p* values that corresponded to the resulting clusters (Supplement).

#### Network Analysis

To perform data-driven network analysis, for each subject, time-series were extracted by averaging voxel time-series within each of equal-sized cortical and subcortical defined regions (nodes) (see Supplement for parcellation template and detailed procedure). Analysis focused on data at frequency interval 0.049–0.101 Hz at 10% cost, which is compatible with prior studies (33). We identified modular community structure, which is a feature of many complex networks, including nervous system. The identification of modules, subsets of nodes densely intraconnected (number of connections between nodes within the module) and sparsely interconnected with nodes in other modules, may uncover functional units (34). The Louvain algorithm (35) as implemented in the Brain Connectivity Toolbox (36) was used to identify modules. Default modularity resolution (gamma = 1) was used in the Louvain algorithm for data presented in the main text and further validated at different gamma levels (Supplement).

## Results

### Functional Striatal Connectivity

Within-group striatal connectivity patterns overlapped with previously described neuro-functional maps. Both groups showed maps of connectivity consistent with models relating the caudate and putamen to cognitive and motor control (4) and the nucleus accumbens to motivational and emotional responses (27) (Supplement, Supplemental Figure S2).

### Between-Group Differences in Striatal Connectivity

Compared with control subjects, OCD patients showed decreased connectivity strength from DCd and PUT to frontal and parietal regions, whereas ventral striatal-frontal connectivity was increased, in line with previous findings (13) (Supplement, Supplemental Figure S3, Supplemental Table S1).

### Cognitive Flexibility and Frontostriatal Connectivity

**Figure 1:**
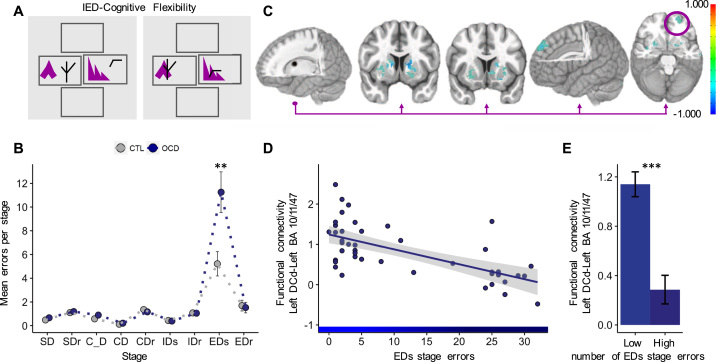
Cognitive flexibility and role of striatal connectivity in obsessive-compulsive disorder (OCD) patients. **(A)** Schematic illustration of the intra-/extradimensional set shift (IED) task testing cognitive flexibility in which stimuli comprising two dimensions (i.e., line and color-filled shape) are presented. **(B)** Mean number of errors by learning stage on the IED task. OCD patients showed impaired cognitive flexibility, evidenced by selectively more errors at the extradimensional shift (EDs) stage compared with matched healthy subjects (CTL). **(C)** Set of brain areas, including left ventrolateral prefrontal cortex (PFC; Brodmann area [BA] 10/11/47), for which significant reduced connectivity with the left dorsal caudate (DCd) was found to be significantly related to worse cognitive flexibility in OCD patients (cluster size after applying a per voxel threshold of *p* < .01; cluster-corrected significance at least *p* < .01). **(D)** The correlation plot shows that reduced functional connectivity between the left dorsal caudate and the left ventrolateral PFC (BA 10/11/47) predicted higher number of errors at the EDs stage in OCD patients. Regression line and 95% confidence interval are shown. **(E)** Bar plot showing mean functional connectivity between the left dorsal caudate and the left ventrolateral PFC (BA 10/11/47) in OCD patients (mean split according to EDs performance). Error bars represent SEM. **p ≤ .01, ***p ≤ .001. CD, superimposed compound discrimination; C_D, separated compound discrimination; CDr, superimposed compound discrimination reversal; CTL, control subjects; EDr, extradimensional shift reversal; IDs, intradimensional shift; IDr, intradimensional shift reversal; SD, simple discrimination; SDr, simple discrimination reversal.

OCD patients exhibited a profound impairment on cognitive flexibility as tested with the IED (Figure 1A). More OCD patients failed to complete all stages of the task (χ^2^_1_ = 7.975, *p* = .005), with patients more likely than control subjects to fail selectively at the EDs stage. All subjects attempted the EDs stage (Supplemental Figure S4). There was a highly significant interaction of stage (IDs, EDs) and group (*F*_1,84_ = 7.128, *p* = .009) in the number of errors. Simple-effect analyses revealed significantly more errors at the EDs stage (*t*_84_ = −2.649, *p* = .01) in OCD patients than control subjects, but no difference at the IDs stage (*t*_84_ = 0.742, *p* = .460) (Figure 1B). Thus, OCD patients were able to form an attentional set and generalize to new stimuli as shown by intact performance up to the EDs stage, but they were selectively impaired when they had to shift attention to a previously irrelevant dimension. There was no significant correlation between the number of errors at the EDs stage and any of the clinical scales, including symptom subtypes and depression severity (all *p* > .203).

To test whether functional connectivity in specific frontostriatal circuits predicted patients’ cognitive flexibility, we used number of errors at the EDs stage as a covariate of interest in the connectivity maps generated from DCd and PUT. In OCD patients, a higher number of errors at the EDs stage was associated with reduced functional connectivity between the left DCd and a set of brain regions, including caudate and putamen bilaterally, right medial frontal gyrus (Brodmann area [BA] 9), and a cluster peaking at BA 10 and extending to the left lateral PFC (BA 10/11/47) (Figure 1C). Specifically, for the left BA 10/11/47, lower connectivity with the left DCd was strongly associated with impaired cognitive flexibility in the OCD sample (Figure 1D, Supplement) and when including control subjects as well (Supplement). Covariation for age and verbal IQ did not alter the results (*p* < .001). Mean split of OCD patients according to the number of EDs stage errors showed that patients severely impaired in cognitive flexibility (high number of EDs stage errors) had significantly reduced functional connectivity between left DCd and left BA 10/11/47 compared with OCD patients with better performance (*t*_42_ = 5.338, *p* < .001) (Figure 1E); the two subgroups were, however, indistinguishable in terms of severity on any of the clinical scales. Similar results were found when testing functional connectivity from the right DCd (Supplemental Table S2) but not when testing functional connectivity from left and right PUT and NAc (Supplemental Table S2, Supplemental Figure S5), revealing the specific relevance of caudate connectivity to cognitive flexibility in OCD patients.

### Goal-Directed Planning and Frontostriatal Connectivity

**Figure 2:**
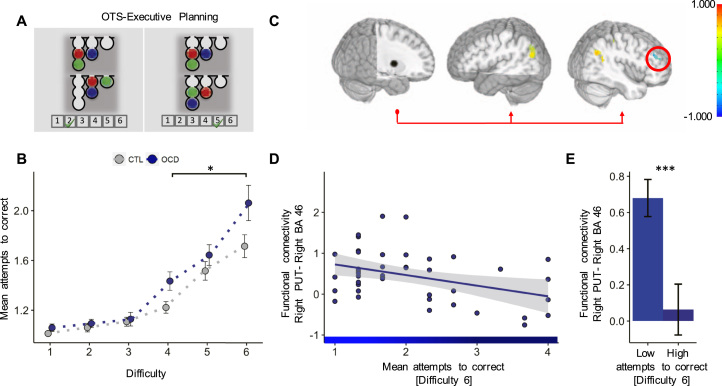
Goal-directed planning and role of striatal connectivity in obsessive-compulsive disorder (OCD) patients. **(A)** Schematic illustration of the One-Touch Stockings of Cambridge (OTS) task testing executive planning. Examples from easy (2 moves) and difficult (5 moves) problems are shown. **(B)** Mean number of attempts to reach correct solution at different difficulty levels on the OTS task. OCD patients show impairment in goal-directed planning compared with matched control subjects (CTL) by requiring more attempts to reach the correct solution at the hard levels of difficulty; there was no group difference for the easy problems. **(C)** Set of brain areas, including right dorsolateral prefrontal cortex (PFC; Brodmann area [BA] 46), for which significant connectivity with the right putamen (PUT) was found to be significantly related to goal-directed executive planning in OCD patients (cluster size after applying a per voxel threshold of *p* < .01; cluster-corrected significance at least *p* < .01). Blue and yellow coloration for weakened and increased connectivity predicting worse or better performance, respectively. **(D)** The correlation plot shows that reduced functional connectivity between the right PUT and the right dorsolateral PFC (BA 46) predicted higher number of attempts at the most difficult level of goal-directed planning (6 moves) in OCD patients. Regression line and 95% confidence interval are shown. **(E)** Bar plot showing mean functional connectivity between the right PUT and the right dorsolateral PFC (BA 46) in OCD patients (mean split according to OTS performance at the most difficult level). Error bars represent SEM. *p ≤ .05, ***p ≤ .001.

**Figure 3:**
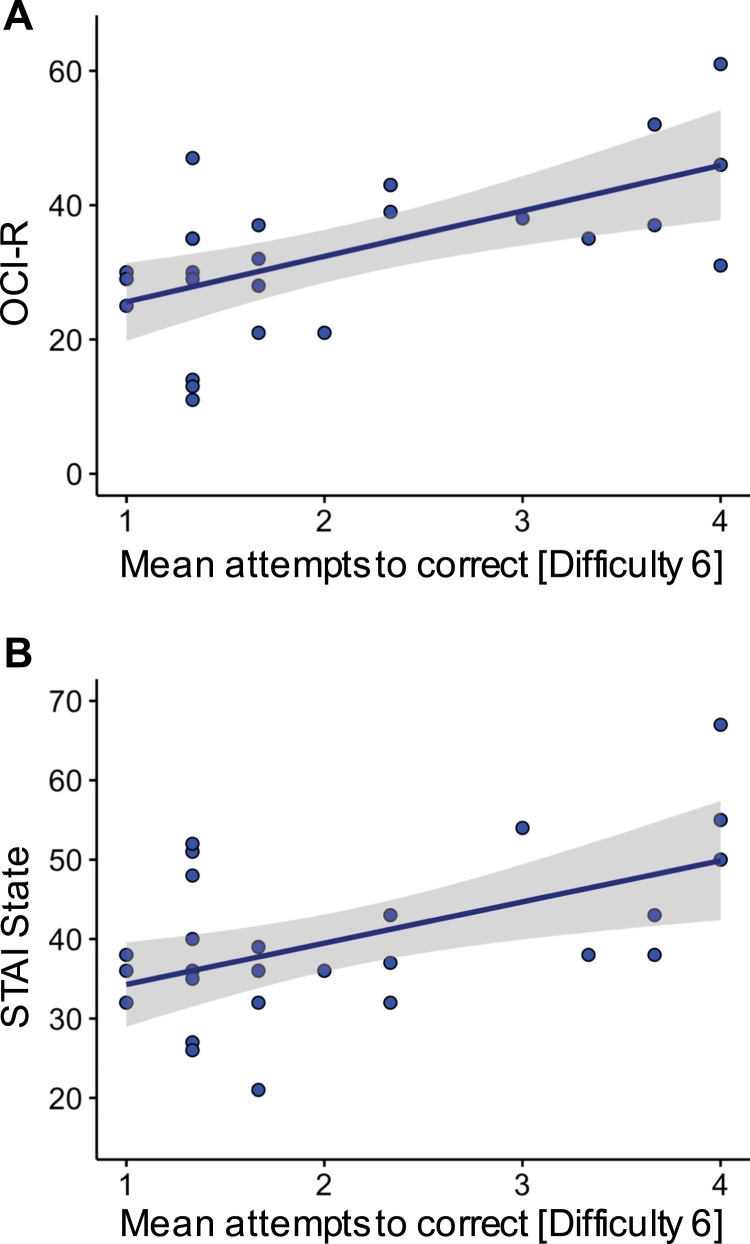
Relation between obsessive-compulsive disorder (OCD) severity and goal-directed performance. Impoverished goal-directed performance at the hardest level of goal-directed planning positively correlated with **(A)** self-reported severity of OCD symptoms (OCI-R: *r*_24_ = .6, *p* = .001, surviving Bonferroni correction) and **(B)** anxiety (STAI-State: *r*_24_ = .531, *p* = .005, surviving Bonferroni correction) in OCD-medicated patients. OCI-R, Obsessive-Compulsive Inventory-Revised (59); STAI, State-Trait Anxiety Inventory (61).

OCD patients showed impaired goal-directed planning abilities at the hard levels of difficulty as tested with the OTS (Figure 2A) and indexed by the increased number of attempts to obtain the correct response (*t*_83_ = −2.427, *p* = .017; Figure 2B) compared with control subjects. There was no group difference for the easy problems. In the medicated patients, increased self-reported severity of OCD symptoms and anxiety positively correlated with poor goal-directed performance at the hardest level of difficulty (Obsessive-Compulsive Inventory-Revised: *r* = .6, *p* < 0.001; State-Trait Anxiety Inventory-State: *r* = .531, *p* < .005, both surviving Bonferroni correction) (Figure 3).

Functional connectivity within a specific frontostriatal circuit predicted patients’ goal-directed planning ability. A higher number of attempts at the most difficult level of the task (6 moves) was associated with reduced functional connectivity between the right PUT and the right dorsolateral PFC (BA 46). In addition, a positive relation was found for the angular gyrus bilaterally (BA 39), whereby better performance was associated with increased functional connectivity from the right PUT (Figure 2C, Supplemental Table S3). Specifically, for the right BA 46, lower connectivity with the right PUT was strongly associated with poor goal-directed planning in the OCD sample (Figure 2D, Supplement) and when including control subjects as well (Supplement). Covariation for age and verbal IQ did not alter the results (*p* = .007). A mean split according to mean number of attempts revealed significantly reduced functional connectivity between the right PUT and right BA 46 in severely impaired patients (*t*_41_ = 3.599, *p* < .001) (Figure 2E). Those patients also exhibited increased anxiety (*t*_41_ = −2.126, *p* = .04) compared with patients performing better on the task. These results were specific to the PUT and not to DCd or NAc (Supplemental Table S3), revealing the specific relevance of putaminal connectivity to goal-directed executive planning in OCD patients.

### Clinical Scores and Ventral Striatal Connectivity

In OCD patients, NAc connectivity was related to clinical scores of anxiety and depression (Supplemental Table S4, Supplement).

### Network Modularity

**Figure 4:**
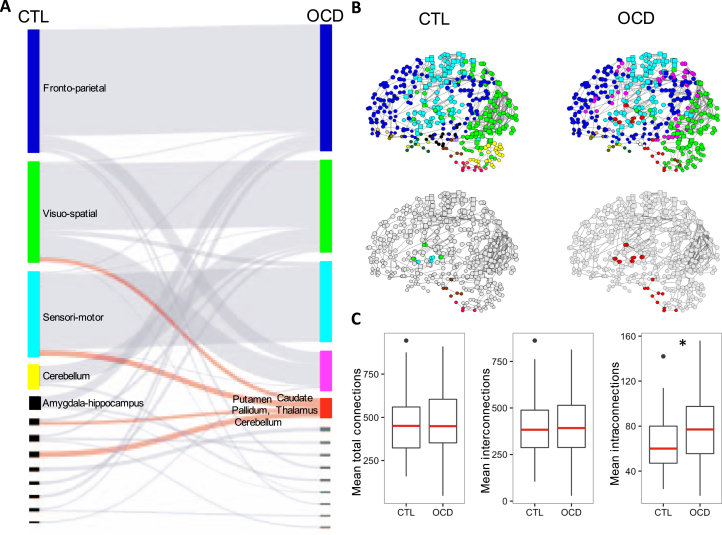
Network modular organization in obsessive-compulsive disorder (OCD) and healthy subjects (CTL). **(A)** Differences in modular organization in OCD and healthy volunteers represented by an alluvial diagram. Each module is separated by white gaps. The flows indicate the nodes for which community structure changes as a function of diagnosis. Red-highlighted module in OCD patients correspond to nodes of the basal ganglia and cerebellum. These nodes are clustered in one module on their own in OCD patients; the same nodes are integrated within separate large cortico-subcortical modules in CTL. **(B)** Nodes for OCD and healthy subjects in anatomical space, color-coded according to module membership. The size of the nodes depends on their number of connections. Respectively, for CTL and OCD patients, lower panels highlight the nodes identified by the modularity algorithm as being part of an independent functional unit in OCD patients (and corresponding to basal ganglia and cerebellum). Nodes are colored according to module membership, highlighting that nodes corresponding to different parts of the basal ganglia (caudate, putamen) and cerebellum are clustered in one single module in OCD patients. In contrast, in healthy subjects the same brain areas are integrated within separate modules. **(C)** Box plot summarizing mean number of connections for nodes identified as being part of an independent functional unit in OCD patients and corresponding to the basal ganglia and the cerebellum. For those nodes, there were no group differences in total number of connections or in the number of interconnections. However, they were significantly more intraconnected in OCD patients than in healthy CTL. *p ≤ .05.

Data-driven network analysis disclosed that, in the OCD sample, nodes corresponding to caudate and putamen as well as the cerebellum were clustered together in a single module (Supplemental Table S5), suggestive of a cohesive functional unit. In contrast, in control subjects, the same striatal and cerebellar regions were distributed among large cortico-subcortical modules (Figure 4A, B). For all the participants, we computed number of connections for each of these nodes; in OCD patients these nodes were more strongly connected to one another (intraconnections) compared with control subjects (*t*_85_ = 2.029, *p* = .046), with no difference for the total number of connections (*t*_85_ = 0.338, *p* = .736) or for number of connections directed toward other nodes (interconnections) (*t*_85_ = 0.049, *p* = .9614) (Figure 4C). Although modularity algorithms are nondeterministic, clustering of these nodes in the same module in OCD and their splitting among different modules in the network of healthy volunteers was robustly observed over multiple runs and several implementations of the analysis (Supplement, Supplemental Figure S6). There were no correlations between network modularity measures and cognitive or clinical measures that survived multiple comparisons (Supplement).

## Discussion

A double dissociation of cognitive deficits contributing to candidate endophenotypes in OCD of goal-directed behavior and cognitive flexibility was identified for separate frontostriatal circuits. A selective deficit in cognitive flexibility (attentional set-shifting) in OCD was associated with reduced functional connectivity between the ventrolateral PFC and the DCd, but not the PUT. In contrast, impaired goal-directed planning was associated with reduced functional connectivity between the dorsolateral PFC and the PUT, but not the DCd. The latter deficit predicted severity of self-reported OCD symptoms and anxiety state and is consistent with recent hypotheses proposing fundamental goal-directed learning impairments in OCD (12). Data-driven network analysis provided evidence in OCD patients of denser connectivity within a group of nodes, including the caudate and putamen, as well as the cerebellum.

Considerable evidence has shown the ventrolateral PFC to be necessary for attentional set-shifting. Excitotoxic lesions of lateral PFC in marmosets produced selective impairments in attentional set-shifting, whereas lesions to the orbitofrontal cortex impaired another form of cognitive flexibility, reversal learning (24). In a human fMRI paradigm, attentional set-shifting selectively recruited the ventrolateral PFC (25). Involvement of the striatum in set-shifting however has hitherto been less clear. Healthy volunteers exhibit caudate activation for reversal in rule classification, but not for extradimensional set-shifting (37). However, set-shifting–related cortical activity was mirrored by activity in the caudate nucleus and dorsal thalamus (38). Recently, resting-state functional connectivity between the ventral striatum and a priori-selected dorsolateral PFC was reported to be associated with attentional set-shifting in a large sample of healthy volunteers (39). By contrast, the present study used unbiased whole-brain analysis to demonstrate that functional connectivity between the caudate and ventrolateral PFC was associated with EDs performance in OCD patients (whether or not combined with the control group for this analysis). It is however possible that additional circuitry, including the ventral striatum, may be associated with EDs performance because we did find some associations in the OCD group with this circuit, although these analyses were post hoc and constrained by multiple comparison. It is nevertheless clear that separate circuits relate to cognitive flexibility and goal-directed planning. A limitation of the present study was the insufficient variability in EDs (and also planning) performances in the control subjects to demonstrate the same relation in that group alone.

Our results provide new evidence that, in OCD patients, the inability to switch attention from a previously relevant dimension to form a new attentional set is intimately related to weakened underlying resting-state connectivity between the DCd and a network of brain regions including the ventrolateral PFC. Consistent with previous data, OCD patients formed attentional sets, as indexed by intact performance on discrimination and intradimensional stages, but showed selective impairment in shifting attention between stimulus dimensions (40, 41). We augmented previous investigations by showing this effect to be independent of medication, in agreement with evidence in animals and humans that serotoninergic mechanisms are not implicated in EDs performance (42, 43). The set-shifting deficit was also independent of clinical severity, in agreement with evidence that it may be an endophenotype (14).

A separate circuit was relevant for the ability to attain goals via single-contingency, instrumental response sequences. Reduced functional connectivity between the putamen and the dorsolateral PFC was associated with inferior performance in OCD patients alone, as well as in the combined sample including control subjects. In task-related imaging studies, the dorsolateral PFC has been classically implicated in executive planning together with parietal and cingulate cortices (26). Anatomical data also support our findings; rich reciprocal connections exist between the dorsolateral PFC and the posterior parietal cortex, which project onto overlapping areas of the putamen (44). In OCD patients, a direct positive association was found such that increased functional connectivity between the PUT and the parietal cortex was predictive of better performance. Goal-directed impairment at the hardest planning levels replicated previous data (14), with no differences between medicated and unmedicated patients. Goal-directed planning failures were associated with self-reported symptom severity and anxiety only in medicated OCD patients, probably because their underlying symptoms were more severe and mitigated by medication. Further studies of first-degree unaffected relatives will clarify whether goal-directed impairment represents a state or trait marker for OCD (45).

Our whole-brain network analysis revealed that, in OCD patients but not in control subjects, nodes belonging to the basal ganglia and cerebellum were more strongly intraconnected, thus corresponding to a unit or “conglomerate.” Results were consistent with previous investigations revealing a higher degree of local connectivity for the putamen in OCD patients (46) and with frequent observations of elevated metabolism of those regions in PET studies during resting state (6). In other words, the stronger intraconnectivity of basal ganglia plus cerebellar nodes drives their separation into a distinct autonomous unit as a function of diagnosis, analogous to that of high impulsive subjects in a comparable study (47). Together with the functional connectivity analysis, this evidence parsimoniously suggests that the PFC exerts less top-down control of these subcortical regions. In general, our complementary imaging analyses suggest that increased connectivity within the striatum might coexist with its decreased functional connectivity to frontoparietal cortical regions, being associated with less flexible and impoverished goal-directed forms of behavior. Although the cerebellum has not hitherto been a major focus of interest in OCD, previous (48, 49, 50) and present findings suggest that it merits further study. In line with anatomical evidence of cerebellar frontostriatal circuitry overlapping (51, 52), we found clustering of striatum and cerebellum in our graph analysis. Much clinical and experimental evidence implicate the cerebellum in executive functions, attentional set-shifting, and motor sequencing (53), consistent with our evidence that striatal-cerebellar connectivity is implicated in attentional set-shifting. We saw no involvement of the cerebellum in relation to planning, perhaps because planning relates to goal-directed rather than habitual/skilled, or what may become just perfect behavior in OCD. Imbalances in corticostriatal connectivity with increased and decreased functional connectivity within the ventral and dorsal striatal networks, respectively, are consistent with previous investigations (13). Existing studies of unmedicated patients, however, only provide mixed findings (54, 55). We directly compared medicated with unmedicated patients, showing that hyperconnectivity of the NAc and PUT to non-PFC cortical areas was more evident in medicated patients. This hyperconnectivity does not necessarily result from medication per se, because the medicated patients might have had more severe underlying symptoms; the hyperconnectivity may therefore reflect OCD symptoms.

PFC regions found here to be functionally connected to basal ganglia and relevant for specific functions, namely ventrolateral PFC for cognitive flexibility and dorsolateral PFC for goal-directed planning, overlapped with those observed in corresponding task-related fMRI studies (25, 26). This adds to the burgeoning literature establishing a close relation between resting-state and task-related functional connectivity in a healthy population (56), and with findings of resting-state functional connectivity related to response inhibition in an OCD population (57). Despite specific neural changes to be expected during the execution of the task, connectivity at rest appears to represent a functional predisposition that enables task execution. Indeed, our results suggest that the observed behavioral deficits depend on this trait-like predisposition, associated with objective behavioral measures heralding symptom manifestation. Thus, resting-state connectivity might serve as a biomarker predicting individual variability in behavioral performance, as shown here. Resting state is a promising practical tool, considering its relative ease and simplicity of data collection and its potential for providing reliable brain mapping from relatively short durations of scanning. We suggest that coupling of well-standardized behavioral indices and brain measures derived from an easily applicable resting-state sequence is a valuable approach for identifying neurobehavioral markers for severe psychiatric disorders, in line with the recent Research Domain Criteria initiative aimed at identifying the biological bases of mental disorders (58).

In conclusion, the present data represent the first identification of discrete striatal-cortical circuits associated with key cognitive endophenotypes for OCD. The data extend the neurobiological model of OCD centered on dysfunction of frontostriatal brain circuits (11). Our study shows that these alterations have a direct link to cognitive processes severely impaired in OCD and of relevance for its symptoms and diagnosis.
