# Supplementary material for: Specific Frontostriatal Circuits for Impaired Cognitive Flexibility and Goal-Directed Planning in Obsessive-Compulsive Disorder: Evidence From Resting-State Functional Connectivity
Source: Biol Psychiatry. 2017 Apr 15;81(8):708–17. doi: 10.1016/j.biopsych.2016.08.009 (PMC6020061; doi:10.1016/j.biopsych.2016.08.009)
Supplement: Supplementary file 1 — Supplementary material [file mmc1.pdf]

Specific Fronto-Striatal Circuits for Impaired Cognitive Flexibility and Goal-Directed Planning in Obsessive-Compulsive Disorder: Evidence From Resting-State Functional Connectivity

Supplemental Information

SUPPLEMENTAL METHODS AND MATERIALS

Participants

Patients were recruited through clinical referral from local psychiatric and psychological services or local advertisement. Control subjects were recruited from the community; none of them were on psychiatric medication and they never suffered from a psychiatric disorder. We ensured that patients met criteria for OCD diagnosis and did not suffer from any current comorbid Axis I disorder. Exclusion criteria for all participants were current substance dependence, head injury and current depression, indexed by Montgomery-Åsberg Depression Rating Scale (MADRS, (1)) exceeding 16. A fully certified consultant psychiatrist, or a consultant psychiatrist when recruitment was conducted through advertisement, made DSM-V diagnoses using an extended clinical interview, supplemented by the Mini International Neuropsychiatric Interview (2). OCD patients were not enrolled in the study if they scored less than 12 on the Yale-Brown Obsessive-Compulsive Scale (YBOCS, (3)) and if they reported hoarding symptomatology. In spite of the imposed a cut-off of maximum score of 16 on MADRS during screening, on the day of testing, one patient reported MADRS scores in excess of this cut-off, scoring 21. We included this patient in the main analyses, as inclusion or exclusion did not affect the main findings. Self-reported measures of anxiety were collected using the State-Trait Anxiety Inventory (STAI, (4)); and, in addition to YBOCS scores, self-reported measures of OCD symptomatology were collected using the Obsessive Compulsive Inventory-Revised (OCI-R, (5)). Twenty-six of the

27 medicated patients were taking selective serotonin reuptake inhibitors (SSRIs), 21 were taking SSRIs in isolation, 4 in combination with tricyclic antidepressants (TCAs) (N=1), atypical antipsychotic (N=2) or both (N=1). The one remaining patient not taking SSRIs was taking a tricyclic antidepressant, clomipramine. All medicated patients were taking stable doses of medication for a minimum of 8 weeks prior to taking part in the study. Seventeen unmedicated patients were also included in the study, being either drug-naïve (n=12) or off medication (n=5) for at least 8 weeks prior taking part of the study (1 patient ceased medication 1 year before inclusion in the study; 1 patient ceased medication 1.5 years before inclusion in the study; 1 patient ceased medication 9 weeks before inclusion in the study; 1 patient ceased medication 3 years before inclusion in the study; 1 patients ceased medication 9 months before inclusion in the study). There was no difference between medicated and unmedicated patients in clinical or demographic measures (all $p>0.165$) including YBOCS scores. However, medicated patients were on stable doses of SSRIs and we expect pharmacological treatment to have long-term efficacy (6; 7), it is plausible to infer that underlying symptomatology of medicated patients is more severe. The study was approved by the Local Research Ethical Committee at the University of Cambridge. Participants were reimbursed for their time and informed consent was obtained prior participation.

Procedure

Imaging Parameters. Data were acquired with a Siemens Trio 3T MRI Scanner and a 32-channel receive-only head coil (Siemens Medical Solutions). Functional images were acquired with a multi-echo planar imaging sequence with online reconstruction [TR, 2.47 s; flip angle, 78°; matrix size, 64 × 64; in-plane resolution, 3.75 mm; FOV, 240 mm; 32 oblique slices, alternating slice acquisition slice thickness 3.75 mm with 10% gap; iPAT factor, 3; bandwidth (BW) = 1,698 Hz/pixel; TE, 12, 28, 44, and 60 ms] (8). Anatomical images for warping and supplemental structural analysis were acquired using a T1-weighted

magnetization prepared rapid gradient echo (MPRAGE) sequence [176 × 240 FOV; 1-mm in-plane resolution; inversion time (TI), 1,100 ms]. Anatomical images were skull-stripped and anatomical and functional images were co-registered and nonlinearly warped to the MNI 152 template.

Resting state acquisition. We measured degree of alertness by asking participant to complete the Stanford Sleepiness Scale (9), which was projected on the screen immediately before and after the acquisition of resting state data. Participants made their answers verbally via a two-way intercom. Participants maintained wakefulness throughout the acquisition of resting state data. Acquisition of the resting state increased subjective sleepiness (F=4.955, df=1,85, p=0.029), with slightly higher scores after (mean=3.03, SD=1.57) than before (mean=2.71, SD=1.41) the acquisition of resting state data. However, the absence of an interaction with group (F=1.196, df=1,85, p=0.277), excluded that observed differences in resting state functional connectivity might be due to different levels of arousal across participants. None of the participants fell asleep during the acquisition of the data.

Behavioral paradigms. The CANTAB Intra/Extra Dimensional Set Shift (IED) is a nine-stage visual discrimination task where stimuli constituted by either one or two artificial dimensions (i.e., colour-filled shapes and lines) are presented. Two stimuli are displayed at a time (10). Reinforcing feedback is provided automatically by the computer so that the subject can learn which stimulus is correct. Initially, simple stimuli, made up of just one of the two dimensions are presented. Subsequently, multidimensional stimuli are introduced, namely lines superimposed on colour-filled shapes. The rule for correct responding is modified at the start of each stage. The participant first learns a series of discriminations in which the same stimulus dimension is relevant and likely to promote the development of an attentional set towards that dimension (e.g., shapes). The Intra Dimensional Shift (IDs) stage involves the transfer of a rule within the same dimension (e.g., shapes) requiring the generalisation of the

learnt set to novel stimuli. Critically, the Extra Dimensional Shift (EDs) stage is the core component, testing cognitive flexibility and requires a transfer of attention to the previously irrelevant dimension (e.g., lines rather than shapes). To pass each stage, six consecutive correct responses are required within 50 trials otherwise the task ends. Subjects who failed to pass a stage were excluded from data analysis for subsequent stages not attempted. The CANTAB One Touch Stockings of Cambridge (OTS) is derived from The Tower of London task (11). As a classic test of executive planning, the participant is required to identify the number of moves necessary to match balls in the lower arrangement to balls in the top half of the display. Problem difficulty varies from 1 to 6 and participants have to work the solution in their head and then touch the correct box at the bottom of the screen to indicate their response. Participants completed other behavioral tasks, unrelated to the present study, and for which analysis is ongoing. The findings reported in our manuscript only refer to specific *a priori* hypotheses and are not the results selected from a larger number of brain-behavior measures. Collection of imaging and behavioral data was conducted on the same day for the large majority of the participants or within a 2 week time interval. Behavioral data for IED and OTS task from one control subject were lost due to technical error. Data from one medicated patient on the OTS were not collected because the patient was tested on the same task in a previous experimental session.

Imaging Preprocessing and Analysis. Raw images for each subject were optimally combined using T2*combination of echoes. For each subject, decomposition into independent components is achieved using FastICA. The identified components are then classified as being BOLD or non-BOLD signal based on physical principles (8) according to which BOLD signal has percent signal changes that are linearly dependent on echo-time, a characteristic of the T2* decay (12; 13). Indeed, percent signal changes of resting BOLD fluctuations demonstrate linear TE dependence according to previous studies (14). Thus

linear TE-dependent percent signal changes is a distinctive characteristic of BOLD T2* signal. Independent component analysis is applied to identify components. On the basis of BOLD linear dependence with echo time, two complementary indexes are used to classify the components, namely a pseudo-F-statistic k and a pseudo-F-statistic r. In other words, components that scale strongly with echo time, will have high k scores, while non-BOLD signal changes will have high r scores. Thus, denoising of the data relies on removing low k / high r components, which represent all non-BOLD signals including motion artefacts. Thus, sources of variance deriving from motion, physiological and scanner artefacts which do not scale with echo-time is discarded in a robust, unsupervised manner. In line with previous reports (13) we used ME independent components regressions to achieve tailored denoising for each subject. This is possible as components for each subject are known and we used them to normalize correlation values. This normalization controls for variability due to varying BOLD sensitivity with subject motion. In order to overcome the stochastic component intrinsic to ICA, the step related to FastICA was run 11 times for each subject. For each subject, we conservatively selected the run which retained the lowest number of BOLD components, those run were then entered in the group analysis and the associated number of components appropriately used as degrees of freedom. Median split of the main cohort of 87 subjects according to a measure of total motion computed as the sum of framewise displacement (FD) (13), confirmed that subject motion reduced the number of BOLD components retained; significantly fewer components were identified for the high motion group (t=-3.941, df=85, p=0.0002).

No band-pass filtering nor smoothing were applied. ME-ICA denoised data were entered in 3dGroupInCorr to estimate functional connectivity. 3dClustSim was used to control for the probability of false positives at p<0.01 at cluster level after applying a per voxel height threshold of p<0.01. We used a Gaussian filter which had equal

smoothing length of 3 mm for each axis and specified the spatial domain using a dataset mask resulting in a grid size of 62x78x67.

Network Analysis. To define regional nodes or parcels of cortex for network analysis, gray matter areas were defined using FSL's cortical Harvard-Oxford probabilistic atlas thresholded at 25%. Voxels were downsampled to n=471 smaller contiguous regions (nodes) with approximately homogeneous sizes (15; 16). Regional time series were only included in further analysis if good quality fMRI data were available for all subjects; regions exhibiting time series z-score<-1.96 in at least one subject were excluded. Due to susceptibility artefacts at the base of the brain in temporal and cerebellar regions, this criterion excluded 12 regions from consideration leaving a complete dataset of 459 regions for each subject. Thus, the pre-processed dataset consisted of 87 individual matrices of regional BOLD oscillations at each of 459 cortical and subcortical regions. The Maximal Overlap Discrete Wavelet Transform (17) was used to decompose each individual regional mean fMRI time series into 4 scales or frequency intervals (Scale 1, 0.101-0.202 Hz; Scale 2, 0.049-0.101 Hz; Scale 3, 0.04-0.024 Hz; Scale 4, 0.012-0.024 Hz) as implemented in the R-based software library brainwaver (freely downloadable at https://cran.r-project.org/) and obtain wavelet correlation matrices for each participant. Those were thresholded at 10% cost to build a binary graph, where the nodes are the brain regions and the edges are the connections included in the graph. We used the minimum spanning tree to ensure that no nodes were disconnected from the rest of the network and then added further edges in order of decreasing correlation strength to produce binary graphs over a range of connection densities, where connection densities refer to the percentage of all possible edges included. Community structures were detected with the Louvain algorithm (18) on 10% density graph constructed from the sample mean correlation matrix of OCD and control subjects. By maximizing the fraction of the network's edges that are intramodular rather than intermodular, the Louvain algorithm identifies the optimal

community partition, i.e., a subdivision of the network into non-overlapping groups of nodes (19). Similarly to other clustering techniques, the partition structure obtained with the Louvain algorithm may vary from run to run, due to the heuristics in the algorithm. In order to overcome this limitation and to make sure of the strength of the community structure identified we verified the stability of the partition as follow. Firstly, separately for graph constructed from sample mean correlation matrix of controls and OCD, the Louvain algorithm was run 100 times independently. In agreement with the method presented by Lancichinetti and Fortunato (20), the consensus modular decomposition over those 100 runs was obtained. For each group separately, we combined the 100 partitions found by the Louvain algorithm and computed the agreement matrix D, which is a *n*x*n* (n=vertices) matrix, whose entry Dij indicates the number of partitions in which vertices i and j of the network were assigned to the same cluster, divided by the number of repetitions (i.e., 100). We used consensus clustering from the Brain Connectivity Toolbox, to seek a consensus partition of the agreement matrix D of OCD patients and healthy subjects converging to their single representative partition respectively. We plotted the two obtained community structures for OCD and healthy participants in the alluvial diagram (as implemented in RAW, http://raw.densitydesign.org/). Secondly, the Louvain algorithm was run 100 times independently on each of the binary matrices of each individual subjects. We obtained a first-order consensus clustering for each individual subject converging to a single representative partition for each subject. Thus, the representative partitions of each subject were used to compute the agreement matrix D for subjects belonging to the control group and separately of subjects belonging to the OCD group. We used consensus clustering to seek consensus partition over controls and OCD subjects to converge to a single representative partition for the two groups separately.

SUPPLEMENTAL RESULTS

Functional striatal connectivity. First, we identified spatial topography patterns of the different components of the striatum. Briefly, the DCd showed significant connectivity with medial frontal gyrus (BA 8), cerebellum, inferior parietal lobule (BA 40), middle frontal gyrus (BA 10), inferior frontal gyrus extending to BA 47, thalamus, posterior cingulate and middle temporal gyrus. Seeds in the PUT showed significant functional connectivity to the superior frontal gyrus (BA 6), cerebellum, inferior frontal gyrus, precentral and postcentral gyrus (BA 3 and BA 4). Connectivity maps of the NAc shifted to a complementary set of brain regions including anterior cingulate (BA 24/32), mid-orbital gyrus, thalamus and insula (see Figure S2).

Between-groups differences in striatal connectivity. OCD patients exhibited significant reduction in functional connectivity between the left DCd seed and right superior frontal gyrus (BA 10/11), left medial frontal gyrus (BA 6), and left inferior temporal gyrus (BA 20) (Figure S3A and Table S1). Similarly, significantly reduced functional connectivity was found between the right DCd and the left inferior parietal lobule (BA 40), left superior frontal gyrus (BA 9, BA 8, BA 6), and middle temporal gyrus (BA 21) (Figure S3A and Table S1). Patients showed reduced connectivity between the right PUT and superior and inferior parietal lobule (BA 7 and BA 39), middle frontal gyrus (BA 6 and BA 8) and postcentral gyrus. From the left PUT, OCD patients exhibited reduced functional connectivity to the postcentral gyrus (BA 5/3) and increased to the superior frontal gyrus (BA 9) (Figure S3B and Table S1). When seeding from the NAc, increased functional connectivity only was found to the anterior cingulate BA 32 in OCD patients (Figure S3C and Table S1). Covariation for age did not change any of these results. Status of the medication only affected connectivity from the right PUT and NAc, and not DCd, bilaterally by increasing functional connectivity to non-PFC cortical regions (Table S1).

Cognitive performance and striatal connectivity. For cognitive flexibility, lower connectivity between the left BA 10/11/47 and the left DCd was associated with impaired cognitive flexibility in the OCD sample (r=-0.646, df=42, p<0.001) and when including controls as well (r=-0.478, df=84, p<0.001). For goal-directed executive planning, lower connectivity between the right BA 46 and the right PUT was associated with poor goal-directed planning in the OCD sample (r=-0.393, df=41, p=0.009) and when including controls as well (r=-0.340, df=83, p=0.002).

Clinical scores and ventral striatal connectivity. Previous studies in the literature have shown nucleus accumbens deep brain stimulation in OCD affecting fronto-striatal network connectivity and reduction in ventral striatum hyper-connectivity being related with improvement in the severity of obsessions and compulsions (21). We thus addressed relationships between ventral striatum connectivity and clinical measures (MADRS, depression; YBOCS, severity of OCD symptoms; STAI-trait and STAI-state, anxiety). Increased depression scores were associated with increased functional connectivity between the ventral striatum and a range of brain regions mostly including subcortical areas (caudate and putamen), thus suggesting increased subcortical connectivity being associated with level of depression. Importantly, our OCD patients were free from any comorbid disorder including depression; depression scores measured with MADRS were higher in patients compared to controls but well below a clinical cut-off for depression. These scores might thus represent an epiphenomenon resulting from (and reflecting) severity of OCD symptomatology. Decreased functional connectivity from the ventral striatum to cortical regions including the cingulate cortex (BA 32) was associated with increased anxiety, while a small cluster showed increased connectivity as a function of state-anxiety. We did not find evidence of ventral striatum connectivity being associated with the severity of OCD symptoms. None of these areas showed overlap with the specific circuit relevant for cognitive

flexibility and executive planning. Interestingly, while cognitive scores seem to have a more precise, anatomically and functionally definite relationship with fronto-striatal connectivity, maps observed for clinical scores are rather diffusely spread throughout the brain.

Network analysis. Consistently with findings reported in the main text, when applying consensus clustering separately for graph constructed from sample mean correlation matrix of controls and OCD (described in the methods section), nodes of caudate, putamen and cerebellum were clustered in one module in OCD patients but split into separate modules in the control group (Figure S6). Results hold for different values of gamma which is a parameter defining the resolution of the modularity structure (Figure S6 A-C). Similarly, when applying consensus clustering on each of of the binary matrices of each individual subject, consistently with findings obtained on the averaged networks, nodes of caudate, putamen and cerebellum were clustered in one module in the OCD group but were split into separate modules in the control group. We explored a possible relationship between modularity measures and clinical and cognitive scores. We applied two different strategies and reported those results that reached or approached significance. First, we applied consensus clustering on each of the binary matrices for each individual subject, to obtain a unique measure of modularity for each subject to be correlated with clinical and cognitive scores. In the entire sample of OCD and controls, there was a trend for a significant association with anxiety scores (STAI-trait, $r=0.196$, $df=85$, $p=0.069$) and OCD traits ($r=0.200$, $df=85$, $p=0.063$). Secondly, for each subject we computed the number of connections for each of the nodes that resulted to be clustered in one cohesive functional unit (Figure 4A). We thus obtained for each subject the number of connections that were directed towards the same subcortical functional unit incorporating the basal ganglia and cerebellum (intra-connections for each subject, Figure 4D) and correlated these values with the clinical and cognitive scores. In the entire sample of OCD and controls, there was a significant

association with anxiety scores (STAI-trait, r=0.256, df-85, p=0.017), and OCD traits (OCI-R, r=0.222, df=85, p =0.039). In this case, there was also a significant association with depression scores in the OCD sample (MADRS, r= 0.362, df=42, p=0.016). Although increased modularity and increased intra-connections in the subcortical unit seem to bear a relationship with anxiety, OCD symptomatology, and depression, none of these correlations survived corrections for multiple comparisons. None of the correlations between modularity and cognitive scores were significant.

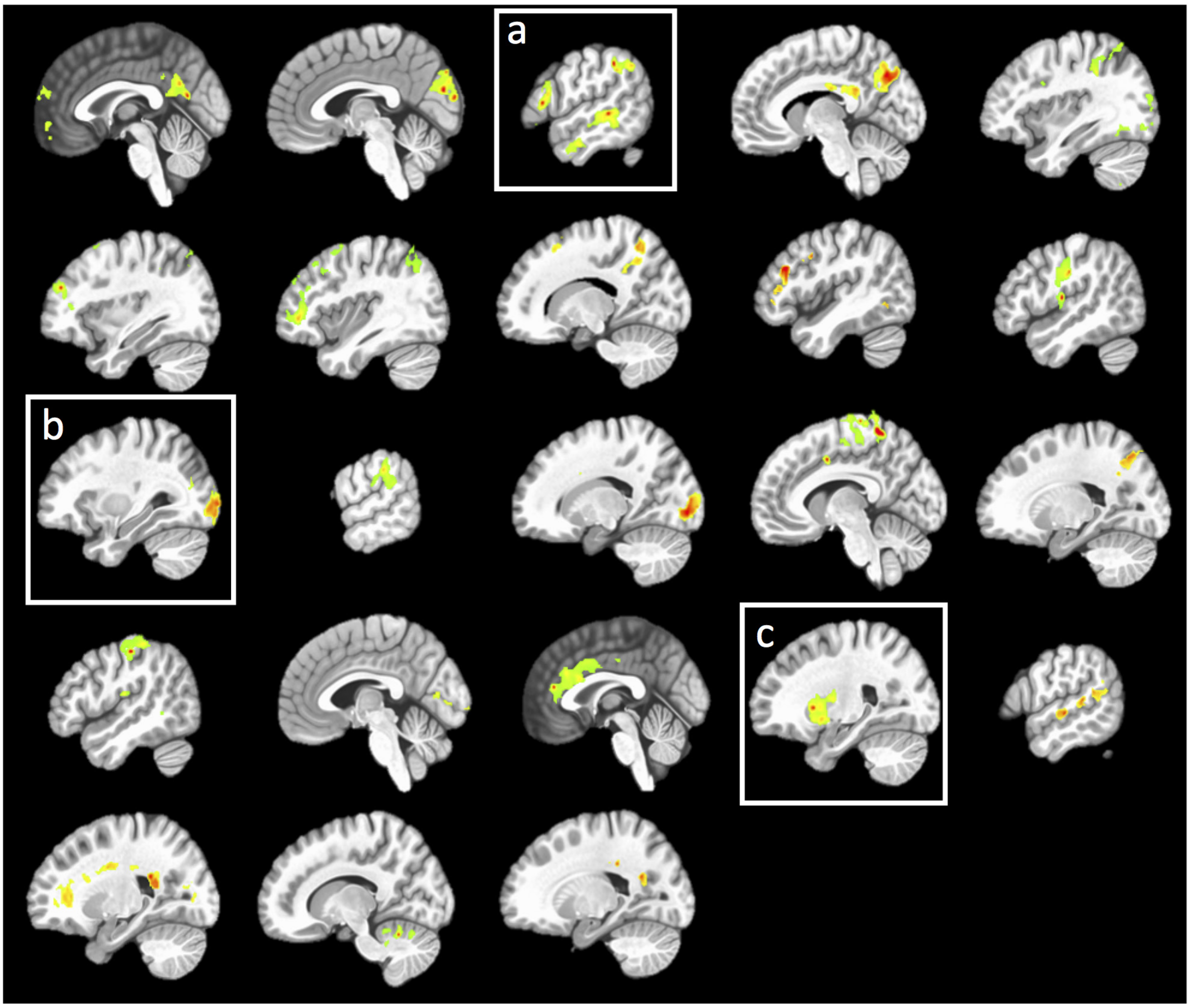

Figure S1. Maps of BOLD components identified by ME-ICA. Maps of BOLD identified by ME-ICA are shown for one representative subject. The components correspond to specific functional areas or anatomically connected networks. Example includes language (a), primary visual (b), and subcortical (c) areas.

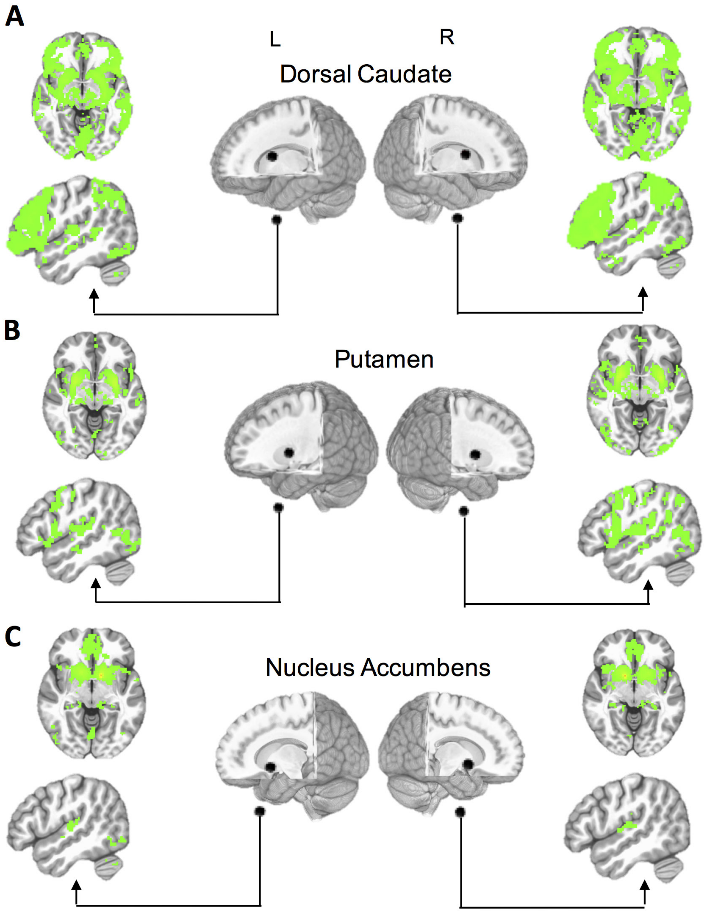

Figure S2. Maps of significant within group functional connectivity. (A) Significant within group functional connectivity in control group from left and right dorsal caudate (DCd) (MNI x y z coordinates: ± 12 6 14). **(B)** Significant within group functional connectivity in control group from left and right putamen (PUT) (MNI x y z coordinates: ± 24 0 3). (**C)** Significant within group functional connectivity in control group from left and right nucleus accumbens (NAc) (MNI x y z coordinates: ± 12 8 -8). Axial and sagittal views are shown.

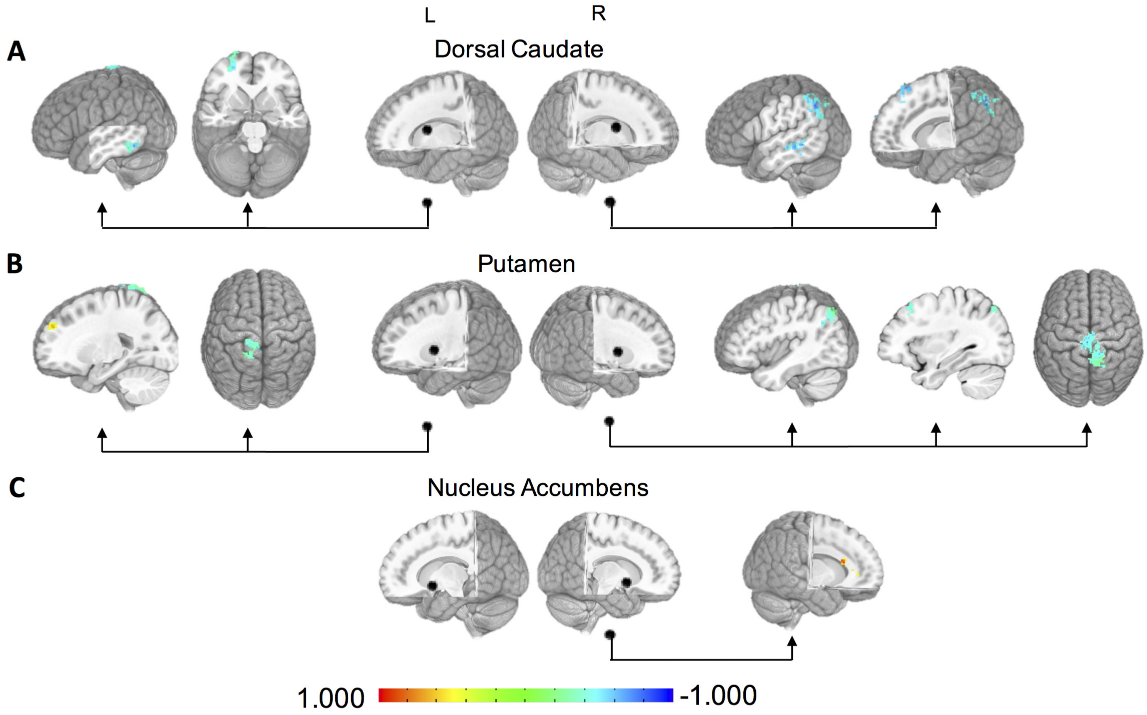

Figure S3. Differences in striatal connectivity between OCD patients and healthy subjects. **(A)** Significant group differences in striatal functional connectivity between OCD patients and healthy subjects when seeding from left and right dorsal caudate (DCd) (MNI x y z coordinates: ± 12 6 14). **(B)** Significant group differences in striatal functional connectivity between OCD patients and healthy subjects when seeding from left and right putamen (PUT) (MNI x y z coordinates: ± 24 0 3). **(C)** Significant group differences in striatal functional connectivity between OCD patients and healthy subjects when seeding from left and right nucleus accumbens (NAc) (MNI x y z coordinates: ± 12 8 -8). Blue to red coloration for decreased and increased functional connectivity, respectively, in OCD patients compared to healthy participants. Seeds and functional connectivity differences are presented in standard neuroanatomical space (MNI152). R, right hemisphere; L, left hemisphere. For visualization purposes, results are displayed at voxel level threshold of $p<0.05$ for those areas showing cluster-corrected significance at $p<0.01$ with a per voxel threshold of $p<0.01$.

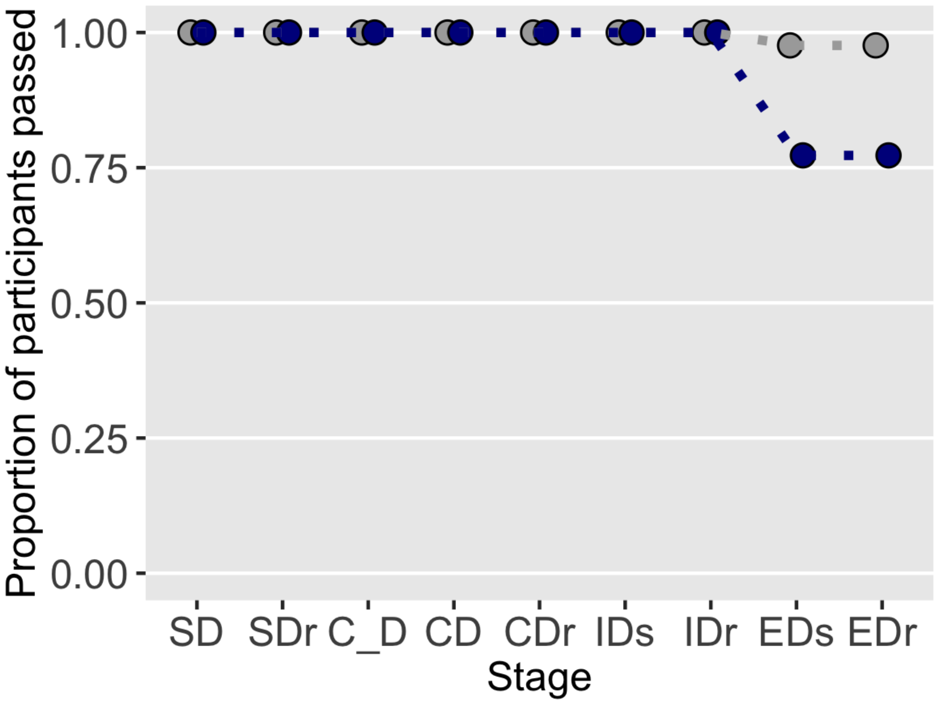

Figure S4. Percentage of subjects passing each stage of the IED task. Percentage of subjects passing each stage shown as a cumulative attrition curve. OCD patients and controls performed similarly up to the Extra-Dimensional shift (EDs) stage where significantly more OCD patients selectively failed.

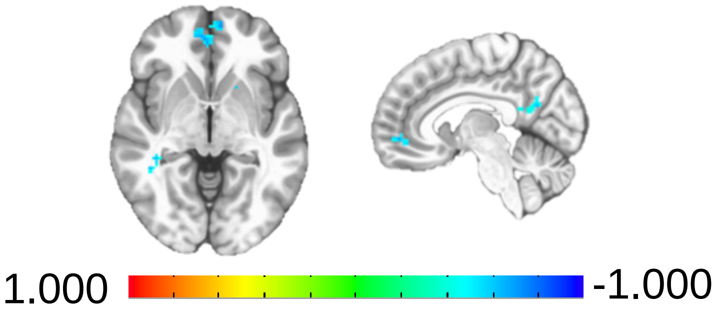

Figure S5. Ventral striatal connectivity and cognitive flexibility in OCD patients. Axial and sagittal view of set of brain areas for which significant reduced connectivity with the right ventral striatum was found to be significantly related to worse cognitive flexibility in OCD patients (cluster-corrected significance p<0.01 with a per voxel threshold of p<0.01).

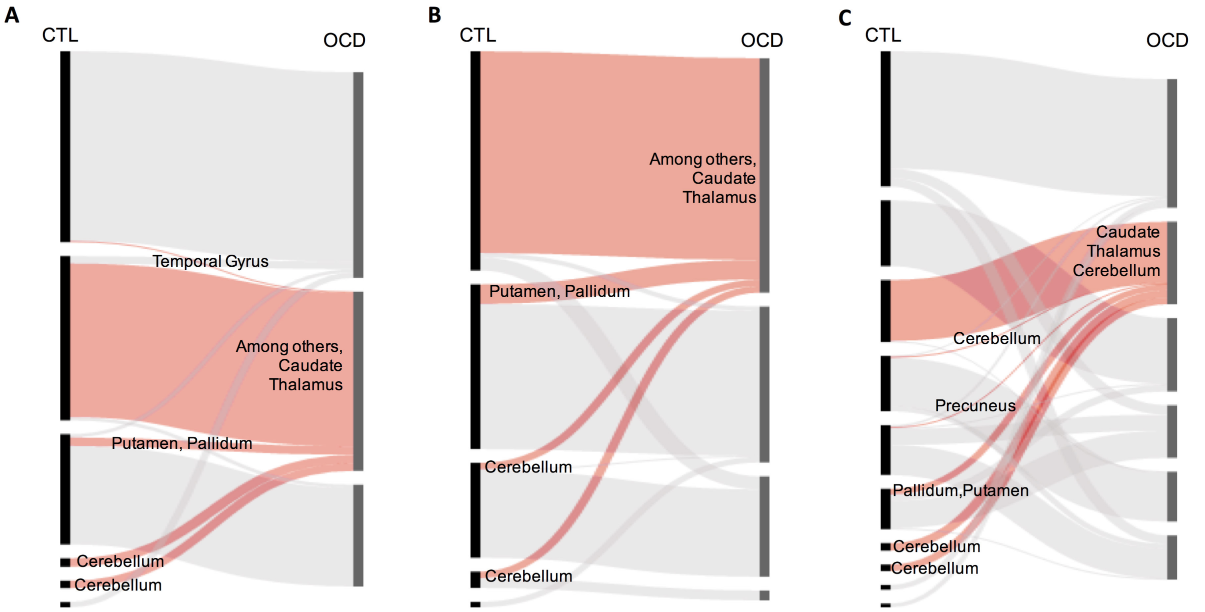

Figure S6. Network modularity structure identified in controls and OCD applying consensus clustering and for increasing levels of gamma resolution. **(A)** Network modularity structure applying consensus clustering and with gamma value equal 1. **(B)** Network modularity structure applying consensus clustering and with gamma value equal 1.2. **(C)** Network modularity structure applying consensus clustering and with gamma value equal 1.5. Across different implementation of the analysis, nodes belonging to caudate, putamen and cerebellum are consistently identified as being part of the same module in OCD patients and split among few modules in healthy volunteers. Differences in modular organization in OCD and healthy volunteers are represented by an alluvial diagram. Each module is separated by white gaps. The flows indicate the nodes for which community structure changes as a function of diagnosis.

Table S1. Group differences in resting state functional connectivity between OCD and healthy subjects

Comparison between OCD and healthy subjects									
**Seed**		**Region**		**BA**	**Ke[a]**	**x**	**y**	**z**	**Direction**
L	DCd	L	Medial Frontal Gyrus	BA 6	47	-6	-30	77	OCD < CTL
		R	Superior Frontal Gyrus	BA 10/11	31	24	54	0	OCD < CTL
		L	Inferior Temporal Gyrus	BA 20	30	-55	-58	-16	OCD < CTL
R	DCd	L	Inferior Parietal Lobule	BA 40	45	-57	-58	42	OCD < CTL
		L	Middle Temporal Gyrus	BA 21/22	33	-59	-32	-7	OCD < CTL
		L	Superior Frontal Gyrus	BA 9	32	-10	54	35	OCD < CTL
		R	Superior Frontal Gyrus	BA 8/6	31	6	40	59	OCD < CTL
		L	Superior Frontal Gyrus	BA 6/8	30	-13	31	63	OCD < CTL
L	PUT	L	Postcentral Gyrus	BA 5/3	45	-15	-46	73	OCD < CTL
		L	Superior Frontal Gyrus	BA 10/9	30	-22	49	31	OCD > CTL
R	PUT	L	Superior Parietal Lobule	BA 7	45	-31	-76	47	OCD < CTL
		R	Postcentral Gyrus		42	13	-46	82	OCD < CTL
		R	Inferior Parietal Lobule	BA 40/39	37	50	-58	40	OCD < CTL
		R	Middle Frontal Gyrus	BA 6/8	37	24	24	63	OCD < CTL
		L	Medial Frontal Gyrus	BA 6	33	-4	-25	77	OCD < CTL
L	NAc	--	--	--	--	--	--	--	--
R	NAc	L	Anterior Cingulate	BA 32	33	-13	21	17	OCD > CTL

Comparison between medicated and unmedicated OCD patients									
**Seed**		**Region**		**BA**	**Ke[a]**	**x**	**y**	**z**	**Direction**
L	DCd	--	--	--	--	--	--	--	--
R	DCd	--	--	--	--	--	--	--	--
L	PUT	--	--	--	--	--	--	--	--
R	PUT	L	Inferior Parietal Lobule	BA 39	74	-41	-76	38	MED > UNM
		L	Thalamus		36	-6	-2	5	MED > UNM
		R	Precuneus		36	3	-67	31	MED > UNM
L	NAc	R	Parahippocampal Gyrus		44	8	-51	-2	MED > UNM
R	NAc	R	Precentral Gyrus	BA 6	56	48	0	-28	MED > UNM
		R	Superior Temporal Gyrus	BA 22	44	57	0	0	MED > UNM
		R	Precentral Gyrus	BA 4/6	43	22	-25	66	MED > UNM

[a] Cluster size after applying a per voxel threshold of $p<0.01$; cluster-corrected significance at least $p<0.01$, coordinates are given in MNI space.

DCd, Dorsal Caudate; PUT, Putamen; NAc, Nucleus Accumbens; L, left; R, right; BA, Brodmann Area.

Table S2. Resting state functional connectivity in cortico-striatal loops in relation to cognitive flexibility

Seed		Region		BA	Ke[a]	x	y	z	Direction
L	DCd	L	Caudate		227	-13	5	7	Negative
		R	Putamen		155	22	0	-7	Negative
		L	Cerebellum (VIIa)		101	-10	-88	-30	Negative
		R	Medial Frontal Gyrus	BA 9	70	1	52	42	Negative
		R	Caudate		54	13	7	7	Negative
		L	Superior Frontal Gyrus	BA 10/11/47	45	-20	61	-4	Negative
		L	Putamen		43	-22	12	-11	Negative
		R	Insula	BA 21/13	34	41	-4	-11	Negative
		L	Cerebellum (VIIa)		31	-24	-74	-32	Negative
		L	Cerebellum (VIIa)		29	-38	-83	-30	Negative
R	DCd	L	Cerebellum (VI)		117	-10	-76	-28	Negative
		R	Inferior Parietal Lobule	BA 40	86	48	-46	54	Negative
		L	Putamen		82	-17	5	12	Negative
		R	Putamen		42	22	0	-9	Negative
		R	Insula	BA 13/21	40	41	-4	-7	Negative
		R	Parahippocampal	BA 28/35	35	22	-18	-9	Negative
		L	Cerebellum (VIIa)		33	-22	-74	-46	Negative
		L	Cerebellum (IX)		32	-6	-55	-35	Negative
L	PUT	L	Putamen		191	-24	-2	3	Negative
		R	Putamen		40	20	3	14	Negative
R	PUT	L	Putamen		232	-22	5	10	Negative
		R	Caudate		70	10	5	10	Negative
		R	Superior Frontal Gyrus	BA 9	49	10	63	19	Negative
		L	Putamen		38	-29	-2	-9	Negative
		L	Superior Temporal Gyrus	BA 22/21	35	-55	-9	3	Negative
		R	Superior Frontal Gyrus	BA 6/8	33	1	19	61	Negative
L	NAc	--	--						
R	NAc	L	Anterior Cingulate	BA 11	68	-8	57	-6	Negative
		R	Posterior Cingulate	BA 31	53	6	-60	21	Negative
		L	Putamen		51	-22	9	-10	Negative
		R	Anterior Cingulate	BA 32	50	4	-49	-4	Negative
		L	Inferior Parietal Lobule	BA 13	41	-45	-46	24	Negative
		R	Caudate		32	38	-31	0	Negative

[a] Cluster size after applying a per voxel threshold of $p<0.01$; cluster-corrected significance at least $p<0.01$, coordinates are given in MNI space.

DCd, Dorsal Caudate; PUT, Putamen; NAc, Nucleus Accumbens; L, left; R, right; BA, Brodmann Area.

Table S3. Resting state functional connectivity in cortico-striatal loops in relation to executive planning

	Seed		Region	BA	Ke [a]	x	y	z	Direction
L	DCd	L	Cerebellum (VIIa)		81	-34	-74	-28	Negative
		R	Precentral Gyrus	BA 4	41	29	-25	56	Positive
R	DCd	--	--	--	--	--	--	--	--
L	PUT	--	--	--	--	--	--	--	--
R	PUT	R	Posterior Cingulate	BA 23	519	10	-51	24	Positive
		L	Angular Gyrus	BA 39	117	-48	-74	26	Positive
		R	Angular Gyrus	BA 39	87	43	-65	31	Positive
		R	Medial Frontal Gyrus	BA 11	73	6	52	-9	Positive
		R	Middle Frontal Gyrus	BA 46	63	43	35	28	Negative
		R	Superior Frontal Gyrus	BA 10	44	22	59	14	Positive
		R	Middle Temporal Gyrus	BA 21	41	64	-4	-14	Positive
		R	Supramarginal Gyrus	BA 39	32	43	-55	21	Positive
		L	Posterior Cingulate	BA 29	30	-10	-46	5	Positive
		L	Fusiform Gyrus	BA 37	29	-29	-41	-16	Positive
		R	Postcentral Gyrus	BA 40/3	29	38	-37	49	Positive
L	NAc	--	--	--	--	--	--	--	--
R	NAc	--	--	--	--	--	--	--	--

[a] Cluster size after applying a per voxel threshold of $p<0.01$; cluster-corrected significance at least $p<0.01$, coordinates are given in MNI space.

DCd, Dorsal Caudate; PUT, Putamen; NAc, Nucleus Accumbens; L, left; R, right; BA, Brodmann Area.

Table S4. Resting state functional connectivity in ventral striatum loop in relation to clinical severity in OCD patients

	Seed		Region		BA	Ke[a]	x	y	z	Direction
**MADRS**	L	NAc	R	Caudate		178	13	24	0	Positive
			L	Putamen		73	27	-17	7	Positive
			L	Putamen		60	-20	17	6	Positive
			R	Posterior Cingulate	BA 30	43	15	-53	12	Positive
			L	Putamen		40	-22	5	-4	Positive
			R	Precuneus	BA 19	40	36	-75	36	Positive
			L	Caudate		32	-17	4	13	Positive
	R	NAc	R	Caudate		277	15	23	-1	Positive
			R	Middle Occipital Gyrus	BA 18	136	34	-90	11	Positive
			L	Putamen		95	-22	14	4	Positive
			R	Fusiform Gyrus	BA 37	79	38	-60	-5	Positive
			L	Cerebellum		51	-36	-48	-35	Positive
			R	Middle Frontal Gyrus	BA 6	47	48	5	41	Positive
			R	Cingulate Gyrus	BA 32	41	10	15	31	Positive
**YBOCS**	L	NAc	--	--						
	R	NAc	--	--						
**STAIT**	L	NAc	R	Precuneus	BA 7	53	6	-58	53	Negative
	R	NAc	L	Cuneus	BA 18	228	-1	-90	14	Negative
			L	Lingual Gyrus	BA 18	204	-13	-74	-4	Negative
			L	Fusiform Gyrus	BA 19	107	-38	-64	-12	Negative
			R	Putamen		100	17	7	-4	Negative
			L	Putamen		94	-22	3	4	Negative
			R	Middle Frontal Gyrus	BA 8	92	34	34	42	Negative
			R	Cingulate Gyrus	BA 32	87	3	22	34	Negative
			L	Putamen/Amygdala		41	-27	-0	-8	Negative
**STAIS**	L	NAc	--	--						
	R	NAc	L	Medial Frontal Gyrus	BA 8	31	-8	17	52	Positive

[a] Cluster size after applying a per voxel threshold of p<0.01; cluster-corrected significance at least p<0.01, coordinates are given in MNI space.

NAc, Nucleus Accumbens; L, left; R, right; BA, Brodmann Area. MADRS, Montgomery-Åsberg Depression Rating Scale; STAIT, State-Trait Anxiety Inventory-Trait; STAIS, State-Trait Anxiety Inventory-State; YBOCS, Yale-Brown Obsessive Compulsive Scale.

Table S5. Brain regions corresponding to an independent functional unit in OCD patients

Side[a]	Region	x	y	z
L	Caudate Nucleus	-14	1	17
L	Caudate Nucleus	-12	14	1
R	Caudate Nucleus	14	3	17
R	Caudate Nucleus	12	16	2
L	Thalamus	-6	-17	3
R	Thalamus	8	-16	3
L	Pallidum	-19	-5	-1
R	Pallidum	20	-4	-1
L	Putamen	-22	8	-2
L	Putamen	-28	-8	3
R	Putamen	22	10	-2
R	Putamen	28	-5	3
L	Cerebellum	-8	-27	-22
R	Cerebellum	6	-32	-21
R	Cerebellum	12	-34	-37
L	Cerebellum Lobule VIIIB	-2	-42	-61
R	Cerebellum Lobule IX	3	-40	-54
L	Cerebellum Lobule IX	-12	-54	-60
L	Cerebellum Lobule X	-4	-39	-37
L	Brain Stem	-2	-18	-30
L	Brain Stem	-7	-27	-39
R	Brain Stem	11	-21	-30
R	Brain Stem	3	-33	-46

[a]Coordinates are given in MNI space, L, left; R, right.

SUPPLEMENTAL REFERENCES

1. Montgomery SA, Asberg M (1979): A new depression scale designed to be sensitive to change. *Br J Psychiatry J Ment Sci*. 134: 382–389.

2. Sheehan DV, Lecrubier Y, Sheehan KH, Amorim P, Janavs J, Weiller E, *et al.* (1998): The Mini-International Neuropsychiatric Interview (M.I.N.I.): the development and validation of a structured diagnostic psychiatric interview for DSM-IV and ICD-10. *J Clin Psychiatry*. 59 Suppl 20: 22–33;quiz 34–57.

3. Goodman WK, Price LH, Rasmussen SA, Mazure C, Fleischmann RL, Hill CL, *et al.* (1989): The Yale-Brown Obsessive Compulsive Scale. I. Development, use, and reliability. *Arch Gen Psychiatry*. 46: 1006–1011.

4. Spielberger CD (1983): Manual for the State-Trait Anxiety Inventory STAI. Palo Alto, CA: Mind Garden.

5. Foa EB, Huppert JD, Leiberg S, Langner R, Kichic R, Hajcak G, Salkovskis PM (2002): The Obsessive-Compulsive Inventory: development and validation of a short version. *Psychol Assess*. 14: 485–496.

6. Koran LM, Hackett E, Rubin A, Wolkow R, Robinson D (2002): Efficacy of sertraline in the long-term treatment of obsessive-compulsive disorder. *Am J Psychiatry*. 159: 88–95.

7. Ravizza L, Barzega G, Bellino S, Bogetto F, Maina G (1996): Drug treatment of obsessive-compulsive disorder (OCD): long-term trial with clomipramine and selective serotonin reuptake inhibitors (SSRIs). *Psychopharmacol Bull*. 32: 167–173.

8. Poser BA, Versluis MJ, Hoogduin JM, Norris DG (2006): BOLD contrast sensitivity enhancement and artifact reduction with multiecho EPI: parallel-acquired inhomogeneity-desensitized fMRI. *Magn Reson Med Off J Soc Magn Reson Med Soc Magn Reson Med*. 55: 1227–1235.

9. Hoddes E, Zarcone V, Smythe H, Phillips R, Dement WC (1973): Quantification of Sleepiness: A New Approach. *Psychophysiology*. 10: 431–436.

10. Downes JJ, Roberts AC, Sahakian BJ, Evenden JL, Morris RG, Robbins TW (1989): Impaired extra-dimensional shift performance in medicated and unmedicated Parkinson’s disease: evidence for a specific attentional dysfunction. *Neuropsychologia*. 27: 1329–1343.

11. Owen AM, Doyon J, Petrides M, Evans AC (1996): Planning and spatial working memory: a positron emission tomography study in humans. *Eur J Neurosci*. 8: 353–364.

12. Kundu P, Inati SJ, Evans JW, Luh W-M, Bandettini PA (2012): Differentiating BOLD and non-BOLD signals in fMRI time series using multi-echo EPI. *NeuroImage*. 60: 1759–1770.

13. Kundu P, Brenowitz ND, Voon V, Worbe Y, Vértes PE, Inati SJ, *et al.* (2013): Integrated strategy for improving functional connectivity mapping using multiecho fMRI. *Proc Natl Acad Sci*. 201301725.

14. Peltier SJ, Noll DC (2002): T(2)(*) dependence of low frequency functional connectivity. *NeuroImage*. 16: 985–992.

15. Craddock RC, James GA, Holtzheimer PE, Hu XP, Mayberg HS (2012): A whole brain fMRI atlas generated via spatially constrained spectral clustering. *Hum Brain Mapp*. 33: 1914–1928.

16. Vértes PE, Alexander-Bloch AF, Gogtay N, Giedd JN, Rapoport JL, Bullmore ET (2012): Simple models of human brain functional networks. *Proc Natl Acad Sci*. 109: 5868–5873.

17. Percival DB, Walden AT (2000): *Wavelet Methods for Time Series Analysis*. Cambridge University Press.

18. Blondel VD, Guillaume J-L, Lambiotte R, Lefebvre E (2008): Fast unfolding of communities in large networks. *J Stat Mech Theory Exp*. 2008: P10008.

19. Newman MEJ (2012): Communities, modules and large-scale structure in networks. *Nat Phys*. 8: 25–31.

20. Lancichinetti A, Fortunato S (2012): Consensus clustering in complex networks. *Sci Rep*. 2: 336.

21. Figee M, Luigjes J, Smolders R, Valencia-Alfonso C-E, van Wingen G, de Kwaasteniet B, *et al.* (2013): Deep brain stimulation restores frontostriatal network activity in obsessive-compulsive disorder. *Nat Neurosci*. 16: 386–387.
